# Elabela in Lipid-Related Cardiometabolic Dysfunction: A Critical Narrative Review

**DOI:** 10.3390/metabo16060408

**Published:** 2026-06-11

**Authors:** Zuzanna Chęcińska-Maciejewska, Ewa Pruszyńska-Oszmałek, Paweł Kołodziejski, Andrzej Ciborek, Hanna Krauss

**Affiliations:** 1 Department of Food and Nutrition, Calisia University, 62-800 Kalisz, Poland; 2 Department of Animal Physiology and Biochemistry, Faculty of Veterinary Medicine and Animal Science, Poznan University of Life Sciences, Wolynska Street 35, 60-637 Poznan, Poland; 3 Faculty of Medicine and Health Sciences, University of Kalisz, 62-800 Kalisz, Poland; 4 Preventive Research Institute, Calisia University, 62-800 Kalisz, Poland

**Keywords:** Elabela, APELA, APLNR, adipose tissue microenvironment, lipid metabolism, insulin resistance, macrophage lipid handling, cardiometabolic biomarker

## Abstract

Elabela (ELA/APELA/Toddler) is an endogenous peptide ligand of the apelin receptor APLNR (also known as APJ) and, together with apelin, forms the apelinergic signalling system. Its role in embryonic development, the cardiovascular system, the kidneys and the endothelium is becoming increasingly well characterised, whilst its function in metabolic regulation remains unresolved. Elabela activates pathways essential for metabolic homeostasis—PI3K/Akt, AMPK-related pathways, redox regulation, inflammatory control and pro-survival cascades—but no study has shown that it directly regulates adipocyte lipid metabolism. This narrative review categorises the evidence at the receptor, organ, immunometabolic and intra-adipocyte levels, and also considers the adipose tissue microenvironment as a distinct level of potential relevance. The available data support a role for Elabela as a candidate mediator of lipid-related metabolic dysfunction—via anti-inflammatory, antioxidant and tissue-protective mechanisms—with macrophage lipid metabolism representing the most informative immunometabolic interface. Human studies remain scarce, heterogeneous and limited by a lack of standardisation in assay methods and the unresolved specificity of isoforms. Elabela should therefore be regarded as a candidate indirect modulator of metabolic homeostasis and a candidate biomarker of cardiometabolic stress or adaptation—not as a confirmed direct regulator of adipocyte lipid metabolism.

## 1. Introduction

The apelinergic system comprises the G protein-coupled receptor APLNR (also known as APJ) and two families of endogenous ligands—apelins and Elabela (ELA/APELA/Toddler) [1]. Both are endogenous agonists of APLNR and together form a pleiotropic signalling system implicated in cardiovascular physiology, fluid homeostasis, embryonic development and—increasingly—in energy metabolism and endocrine regulation [2,3,4,5,6].

Elabela was identified later than apelin, and its essential role in embryogenesis, particularly in cardiovascular development, was initially recognised [1]. Subsequent studies have extended its significance to the functioning of blood vessels, the heart, the kidneys and the endothelium in the adult body [3,7,8]. It is encoded by the APELA gene as a 54-amino acid precursor and occurs in three bioactive isoforms—ELA-32, ELA-21 and ELA-11 [3,8]. In contrast to its developmental and cardiovascular roles, its metabolic role has not yet been unequivocally established [4,9,10].

A central methodological aspect is asymmetry: the evidence base for apelin is significantly stronger than that for Elabela. Effects on lipolysis, obesity and glycaemic regulation have been demonstrated mainly for apelin [7,9,11,12,13], whilst Elabela remains discussed more cautiously as a candidate metabolic modulator [4,9,14]. The overall evidence map is presented in Table 1.

Accordingly, this review critically assesses whether the current literature supports a direct metabolic role for Elabela, particularly in adipose tissue and lipid metabolism. The analytical framework distinguishes direct intra-adipocyte data from indirect evidence, such as mechanistic, tissue-protective, immunometabolic, biomarker-based, and evidence from the apelin literature, and treats the adipose tissue microenvironment as a separate level of relevance (Table 2). This distinction has been operationalised in the section devoted to direct versus indirect evidence (Section 5, Table 3), in the table synthesising clinical data (Table 4), and in the comparative translational table of apelin and Elabela (Section 10, Table 5).

## 2. Methods: Literature Search Strategy and Scope of the Review

This article is a narrative review with a structured approach, rather than a systematic review. The literature was identified through targeted searches in PubMed/MEDLINE, Scopus and Web of Science, supplemented by Google Scholar and iterative “citation snowballing” from source articles and relevant thematic reviews. Snowballing was used as an auxiliary rather than systematic method—as a tool for cross-verification and thematic completeness. The final update of the searches was carried out in April 2026.

Search terms were combined using Boolean operators and included the core terms: “Elabela”, “ELA”, “APELA”, “Toddler”, “apelinergic system”, “apelin receptor”, “APLNR”, “APJ”, combined with terms from the metabolic domain: “adipose tissue”, “adipocyte”, “lipid metabolism”, “lipolysis”, “lipogenesis”, “obesity”, “insulin resistance”, “type 2 diabetes”, “metabolic syndrome”, “macrophage polarisation”, “foam cell”, “atherosclerosis”, “oxidative stress”, “AMPK”, “PI3K/Akt”, “SIRT3”, “biased agonism” and “cardiometabolic biomarker”. Separate searches were conducted for the names of the ELA-32, ELA-21 and ELA-11 isoforms.

The priority period was 2013–2026—from the initial identification of Elabela to the most recent update of the searches. Fundamental papers on the biology of apelin and APLNR published prior to 2013 were included where mechanistically necessary. No initial language restriction was applied; in practice, full-text English-language articles were used. Acceptable publication types included peer-reviewed original experimental studies, peer-reviewed clinical cohort studies and peer-reviewed review articles; editorial articles and commentaries were treated solely as a source of context.

Inclusion criteria focused on metabolic relevance. Studies were included if (i) they concerned Elabela biology in metabolically relevant tissues, (ii) they reported direct experimental effects on adipocytes, hepatocytes, vascular or immune cells relevant to lipid or glucose metabolism, (iii) provided biomarker data in humans with obesity, metabolic syndrome, type 2 diabetes or lipid-related cardiometabolic disease, or (iv) provided mechanistic insights into APLNR-dependent or potentially non-canonical signalling. Studies focusing exclusively on developmental, oncological, pre-eclampsia or purely renal issues were retained only if they provided mechanistic information relevant to the metabolic question. Exclusion criteria included: studies that did not distinguish between Elabela and apelin in the experimental design, non-peer-reviewed sources, and publications that could not be verified bibliographically.

The selection and synthesis were performed by the authors; independent duplicate selection was not undertaken, consistent with the narrative-review design. Cross-verification involved comparing the material with the reference lists of recent thematic reviews concerning the apelinergic system in metabolic and cardiometabolic diseases [6,8]. Following deduplication and eligibility assessment, 37 peer-reviewed sources cited in the text, tables and figure captions were retained. Discrepancies were interpreted descriptively by analysing differences in biological material, assay platform, selected Elabela isoform, study population and disease severity. No quantitative meta-analysis was performed.

No formal tool for assessing the risk of systematic error (e.g., the Newcastle–Ottawa scale, ROBIS, AMSTAR-2) or formal GRADE system was used. The descriptive categories of evidence used in Table 1 and Table 3 constitute a transparent interpretative heuristic rather than a formal qualitative assessment; the synthesis should be read as a structured narrative. The deliberate decision not to apply formal risk-of-bias instruments (Newcastle–Ottawa, ROBIS, AMSTAR-2) or GRADE certainty assessment reflects three considerations specific to the current evidence base on Elabela. First, the body of the available literature is dominated by heterogeneous, mostly small cross-sectional biomarker studies and mechanistic experiments performed in non-adipocyte models, for which the listed instruments—designed primarily for comparative effectiveness, diagnostic accuracy or intervention studies—provide limited discriminatory value. Second, the methodological diversity across biological matrices, ELISA platforms, and isoform specifications (ELA-32, ELA-21, ELA-11) precludes the standardised comparability that formal grading systems presuppose. Third, the principal interpretative risk in this field is not bias within individual studies but inter-study incomparability and the extrapolation of apelin-derived conclusions to Elabela; this risk is addressed throughout the review by explicit separation of direct from indirect evidence (Section 5, Table 3) and by the comparative translational table for apelin and Elabela (Section 10, Table 5). The synthesis should therefore be read as a structured narrative rather than a formally graded assessment.

## 3. Molecular Organisation of the Elabela/APLNR Axis

Elabela is encoded by the APELA gene and undergoes proteolytic processing into shorter bioactive peptides. The precursor is a 54-amino acid protein with a predicted N-terminal signal sequence and a mature form 32 amino acids in length (ELA-32); shorter isoforms—ELA-21 and ELA-11—are formed as a result of cleavage by precursor convertases, including furin-dependent cleavage [1,3,8,26]. At the receptor level, binding of Elabela to APLNR leads to inhibition of cyclic AMP production, activation of ERK1/2, mobilisation of intracellular Ca^2+^ and receptor internalisation [5,26,27,28].

APLNR is a G protein-coupled receptor of class A, widely expressed in endothelial cells, vascular smooth muscle, cardiomyocytes and metabolically active tissues [3,5,7,29]. This distribution suggests that Elabela signalling may influence metabolism both through direct responses of parenchymal cells and via vascular, inflammatory and inter-organ mechanisms [6,7,30].

**Figure 1:**
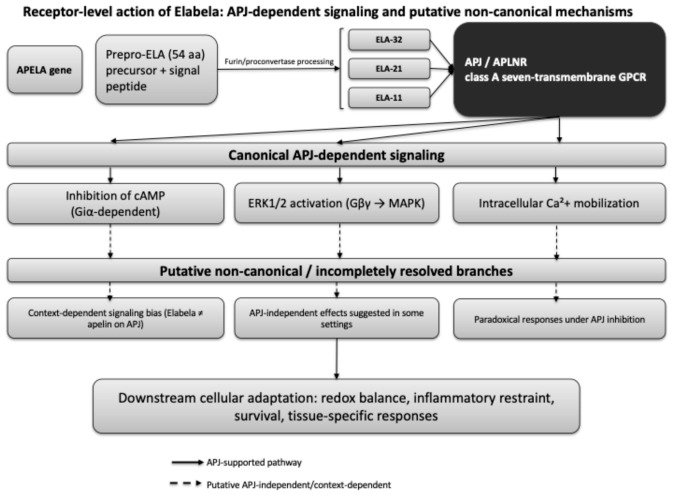
Elabela’s action at the receptor level: APLNR-dependent signalling and potential non-canonical mechanisms. Elabela is derived from a precursor encoded by APELA and is processed into three bioactive isoforms: ELA-32, ELA-21 and ELA-11. Binding to APLNR, a class A seven-transmembrane GPCR, leads to cAMP inhibition (Gαi-dependent), ERK1/2 activation (via Gβγ–MAPK) and intracellular Ca^2+^ mobilisation. As Elabela and apelin differ in their receptor interaction, signal bias balance and downstream efficacy, the diagram also reflects context-dependent signal bias or non-canonical mechanisms. Solid arrows—supported pathways for APLNR; dashed arrows—branches potentially independent of APLNR or not fully resolved. APELA—the gene encoding Elabela; APLNR—the apelin receptor (APJ); cAMP—cyclic AMP; ELA—Elabela; ERK1/2—extracellular signal-regulated kinases 1 and 2; GPCR—G protein-coupled receptor; MAPK—mitogen-activated protein kinase. Furin is a calcium-dependent serine proprotein convertase of the PCSK family, responsible for the proteolytic maturation of protein precursors by cleavage at polybasic motifs, typically R-X-(K/R)-R [31,32,33].

Elabela and apelin share the APLNR but are not functionally interchangeable. Comparative biochemical and pharmacological studies demonstrate distinct signalling profiles for individual apelinergic ligands—Apelin-13, [Pyr1]-Apelin-13, Apelin-17, Apelin-36, ELA-21 and ELA-32—which activate APLNR with varying involvement of G-protein-dependent and β-arrestin-dependent pathways, with ELA-32 exhibiting a particularly strong β-arrestin bias [22,23,25]. The metabolic consequences of Elabela cannot therefore be directly inferred from data on apelin; the implications of this are discussed in Section 10 and summarised in Table 5. The receptor-level organisation and signalling branches are summarised in Figure 1.

## 4. Biological Basis for Elabela’s Role in Metabolic Regulation

The rationale for studying Elabela in metabolic diseases stems from the overlap between APLNR-dependent signalling and cascades critical for cellular homeostasis under conditions of substrate excess, glucotoxicity, lipotoxicity and inflammatory stress. Mechanistic studies consistently identify PI3K/Akt, ERK, AMPK, oxidative stress control and anti-apoptotic signalling as pathways activated downstream of APLNR [4,6,9,24,30].

Adipose tissue is an active endocrine organ whose dysfunction—adipocyte hypertrophy, impaired adipokine secretion, chronic low-grade inflammation, impaired insulin signalling, impaired lipolysis and mitochondrial inefficiency—contributes to obesity, insulin resistance, type 2 diabetes and metabolic syndrome [9,13]. A peptide capable of modulating inflammation, redox balance, mitochondrial integrity or insulin-like signalling is therefore a biologically plausible candidate for modulating adipose tissue metabolism. Biological plausibility does not, however, constitute direct evidence: current support for Elabela’s metabolic role derives primarily from vascular, renal, endothelial and macrophage models [7,21,30,34], rather than from dedicated adipocyte studies—this distinction is formalised in Section 5 and Table 3.

## 5. Direct Evidence vs. Indirect Evidence Regarding the Metabolic Role of Elabela

Given the asymmetry between findings at the receptor, organ, immunometabolic and adipocyte levels, a clear analytical separation is useful. Four layers of evidence can be distinguished, as summarised in Table 3.

The biology at the receptor and ligand level is well documented. APLNR is a class A GPCR, and the canonical Elabela/APLNR effector profile is described in Section 3. Differences in signalling bias between Elabela and apelin have also been documented [22,23,25]. These findings pertain to signalling rather than metabolic physiology as such.

Organ-level and tissue-protective effects are well documented in non-adipocyte systems. Elabela alleviates oxidative stress, inflammation, fibrosis and apoptosis in cardiac, renal and endothelial models relevant to metabolic stress [8,24,34,35]. These effects are mechanistically informative, but do not in themselves establish action at the adipocyte level.

Immunometabolic effects—most extensively documented in atherosclerosis models—constitute a direct experimental link between Elabela and lipid-related pathology, and are described mechanistically in Section 6. These are direct data concerning lipid metabolism in immune cells, qualitatively different from adipocyte data, although both phenomena belong to the broader domain of lipid metabolism.

Direct intra-adipocyte data for Elabela remain insufficient. No study has yet demonstrated that Elabela—at physiologically relevant concentrations—directly regulates lipogenesis, lipolysis, fatty acid oxidation or adipocyte differentiation in mature human or mouse adipocyte systems in a robust and reproducible manner. Claims of direct adipocyte regulation are therefore based on extrapolations from APLNR-dependent signalling biology, from data obtained in immune cells, or from the apelin literature. To avoid any unintended implication of established metabolic effects, the four-level analytical framework outlined above should be read as a hierarchy of evidentiary confidence rather than as a continuum of equivalent support. Receptor-level signalling and tissue-protective effects in non-adipocyte systems are mechanistically documented; immunometabolic effects in macrophages are directly demonstrated within models of vascular lipid pathology; intra-adipocyte effects remain mechanistically plausible but experimentally unconfirmed. Throughout this review, statements describing potential metabolic relevance refer specifically to the indirect and immunometabolic levels and should not be interpreted as evidence of direct adipocyte regulation by Elabela.

## 6. Mechanistic Evidence Relevant to Metabolic Homeostasis

Mechanistic data suggest that Elabela may exert metabolically significant effects via cytoprotective and anti-inflammatory signalling. Particularly informative are studies linking Elabela to the SIRT3/Foxo3a axis, the alleviation of oxidative stress, and AMPK/NLRP3-dependent protection in models of diabetic damage [34,35]; the pathways activated are relevant to metabolic diseases, despite the non-adipocyte experimental context.

SIRT3-dependent regulation suggests an influence on mitochondrial redox balance and resistance to oxidative damage—both of which are relevant to adipocyte dysfunction and systemic insulin resistance [35]. AMPK-dependent effects suggest an interface between cellular energy sensing, inflammation suppression and metabolic adaptation [34]. Anti-apoptotic and anti-inflammatory properties may indirectly protect the function of organs exposed to metabolic stress—including blood vessels, the myocardium, the kidneys, and potentially also adipose tissue [7,8,24,30].

**Figure 2:**
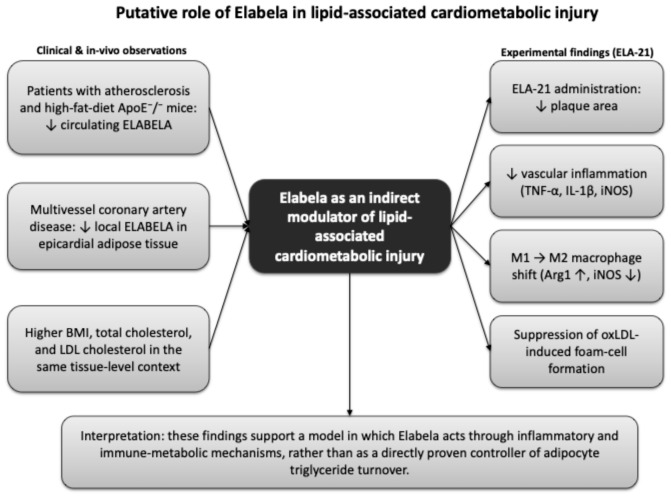
Proposed role of Elabela in lipid-related cardiometabolic damage. The diagram integrates current disease-oriented evidence linking Elabela to lipid pathology. In atherosclerotic conditions, lower circulating concentrations of Elabela are associated with a greater disease burden; local reductions have also been observed in epicardial adipose tissue in patients with multivessel coronary artery disease and in hypertensive populations with increased carotid IMT. Experimentally, administration of ELA-21 reduces plaque area and vascular inflammation, shifts macrophages towards an anti-inflammatory (M2) phenotype, and inhibits oxidised LDL-induced foam cell formation. These findings are consistent with a model in which Elabela is a candidate indirect modulator of lipid damage via inflammatory and immunometabolic mechanisms, rather than a directly proven regulator of triglyceride turnover in adipocytes. Arg1—arginase 1; ApoE^−/−^—apolipoprotein E knockout mouse; BMI—body mass index; ELA—Elabela; IL-1β—interleukin-1β; iNOS—inducible nitric oxide synthase; LDL-C—LDL cholesterol; M1/M2—pro- and anti-inflammatory macrophages; oxLDL—oxidised LDL; TNF-α—tumour necrosis factor alpha.

Data from atherosclerosis models extend this framework to lipid-related pathology. Tang et al. reported reduced plasma Elabela levels in patients with atherosclerosis and in ApoE^−/−^ mice fed a high-fat diet, whilst administration of ELA-21 reduced plaque area, alleviated inflammation, restored the M1/M2 balance towards a more anti-inflammatory state, and inhibited oxLDL-induced foam cell formation [21]. Although this is not an adipocyte model, it is highly relevant to lipid-related pathology, as macrophage lipid metabolism and inflammatory polarisation are key to cardiometabolic diseases. The disease-oriented evidence is integrated in Figure 2.

Collectively, the data support a model in which Elabela influences metabolic diseases primarily by shaping the inflammatory and oxidative environment, rather than by acting as a canonical, direct regulator of triglyceride turnover in the adipocyte.

## 7. Elabela and Adipose Tissue: Current State of Evidence

Adipose tissue is heterogeneous and comprises mature adipocytes, the stromal-vascular fraction (preadipocytes, endothelial cells, pericytes, fibroblasts) and resident macrophages [9,13]. In obesity, the microenvironment undergoes coordinated remodelling: hypertrophy, depletion of capillaries, infiltration and shifts in macrophage polarisation, and changes in paracrine signalling [9,13]. The peptide may therefore influence adipose tissue metabolism via qualitatively distinct pathways.

**Figure 3:**
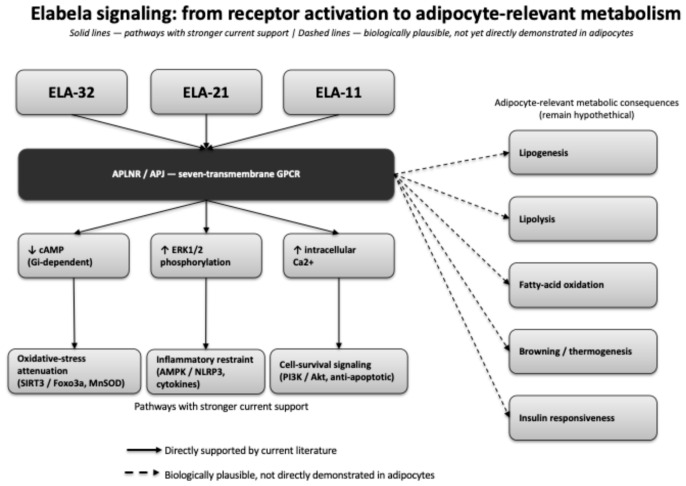
Proposed scheme linking Elabela signalling to metabolic processes relevant to adipocytes. Elabela (ELA-32/ELA-21/ELA-11), acting primarily via APLNR, initiates G-protein-dependent signalling, inhibition of cAMP production, activation of ERK1/2, mobilisation of intracellular Ca^2+^, and the cytoprotective cascades outlined below. Pathways with stronger current evidence—mitigation of oxidative stress, reduction in inflammation and improvement of cellular immunity—have been distinguished from metabolic consequences relevant to adipocytes, which remain hypothetical: changes in lipogenesis, lipolysis, fatty acid oxidation, browning and insulin sensitivity. Dotted arrows indicate biologically plausible pathways, but not directly demonstrated at the adipocyte level. AMPK—AMP-activated protein kinase; APLNR—apelin receptor (APJ); cAMP—cyclic adenosine-3′,5′-monophosphate; ERK1/2—extracellular signal-regulated kinases 1 and 2; MnSOD—mitochondrial superoxide dismutase; NLRP3—pyrin-containing NLR-3; PI3K—phosphoinositide 3-kinase; ROS—reactive oxygen species; SIRT3—sirtuin 3. The figure conceptually separates three analytical levels at which Elabela may act in adipose tissue biology. The intra-adipocyte level refers to direct effects within mature adipocytes—lipogenesis, lipolysis, fatty acid oxidation, mitochondrial respiration, insulin signalling and differentiation—for which direct experimental evidence specific to Elabela is currently insufficient (depicted with dashed arrows). The immunometabolic level refers to Elabela-dependent effects on macrophage polarisation (M1/M2 balance), oxLDL-induced foam cell formation and inflammatory tone of immune cells, for which direct experimental data exist within atherosclerosis models. The microenvironmental level refers to effects across the stromal-vascular fraction, the endothelial–adipocyte interface, and depot-specific paracrine communication, for which biological plausibility derives from the documented APLNR distribution and tissue-protective biology of Elabela. The visual distinction between these levels is intended to make the asymmetry of supporting evidence explicit and to prevent conceptual conflation across compartments.

In this context, five levels of potential relevance can be identified for Elabela: (i) intra-adipocyte effects on lipid metabolism, differentiation or insulin signalling; (ii) effects in the stromal-vascular fraction, including preadipocyte biology and angiogenic remodelling; (iii) effects at the vessel–adipose tissue interface concerning endothelial function and microcirculatory perfusion; (iv) macrophage–adipocyte signalling via inflammatory polarisation and lipid metabolism phenotypes [21]; (v) effects at the microenvironment level of fat depots, differing for subcutaneous, visceral, perivascular and epicardial tissues [16]. This distinction is reflected in Table 3 and conceptually in Figure 3.

Against this background, current evidence does not support strong claims of direct intra-adipocyte regulation by Elabela. There is a lack of robust, direct data regarding Elabela-specific effects on differentiation, lipogenesis, lipolysis, fatty acid oxidation or thermogenic programming in primary adipocytes [4,9,24]. A more plausible interpretation is that any metabolic role of Elabela may concern the adipose tissue microenvironment—particularly the immunometabolic and vascular interfaces—rather than lipid turnover in the mature adipocyte itself. Tissue data from subcutaneous adipose tissue in patients with multivessel coronary artery disease [16] are consistent with this interpretation, but do not replace mechanistic experiments on adipocytes. A more comprehensive interpretation requires explicit integration of adipose tissue biology. Adipose depots are not biologically uniform: visceral adipose tissue exhibits higher metabolic activity, greater inflammatory infiltrate and stronger associations with insulin resistance than subcutaneous adipose tissue, whilst perivascular and epicardial depots have distinct paracrine functions relevant to vascular biology [9,13,16]. In obesity, the immune cell composition shifts towards pro-inflammatory M1 macrophage dominance, T-cell infiltration, and dendritic cell accumulation [9,13,21]. The tissue concurrently undergoes vascular rarefaction with reduced capillary density and impaired angiogenic adaptation, fibrosis-related extracellular matrix remodelling, and a relative loss of thermogenic, UCP1-expressing adipocytes in beige and brown depots [9,13]. The APLNR-dependent biology of Elabela—documented at the endothelial, immunometabolic and cytoprotective levels [7,8,21,24,30,34]—intersects with several of these processes, but the depot-specific, cell type-specific and obesity stage-specific consequences remain experimentally undetermined. The proposed role of Elabela at the microenvironment level should therefore be interpreted as a structured hypothesis spanning these heterogeneous compartments, rather than as a unified adipose tissue mechanism.

## 8. Elabela and Lipid Metabolism

Lipid metabolism is the most clinically compelling, yet also the most prone to overinterpretation, aspect of this topic. Current evidence supports the view that Elabela is not a proven direct regulator of lipid metabolism, but may indirectly influence lipid-related metabolic dysfunction through several overlapping mechanisms: modulation of inflammatory signalling [21,34]; alleviation of oxidative stress [35]; protection of mitochondrial function [35]; regulation of macrophage lipid handling and inhibition of foam cell formation [21]; and tissue adaptation in metabolically stressed environments [7,16]. The asymmetry of evidence for apelin/Elabela is discussed in Section 10 and Table 5.

The atherosclerosis data are particularly instructive. The macrophage-level effects detailed in Section 6 are relevant to lipid pathology, but pertain to immunometabolic mechanisms within vascular disease rather than classical adipocyte lipid metabolism. They therefore constitute evidence of lipid-related pathophysiological relevance, rather than evidence of direct lipolytic or lipogenic control in adipocytes. For terminological precision, the term “modulation of lipid-related metabolic dysfunction” as used throughout this section refers exclusively to indirect mechanisms—anti-inflammatory, antioxidant, mitochondrial-protective and macrophage-related—rather than to canonical regulation of adipocyte triglyceride turnover. Current evidence does not warrant the conclusion that Elabela alters lipogenic or lipolytic flux in mature adipocytes, and the immunometabolic effects documented at the macrophage level should not be conflated with classical adipocyte lipid metabolism.

## 9. Evidence in Humans: Obesity, Metabolic Syndrome and Diabetes

The literature on Elabela in metabolic diseases in humans remains limited, heterogeneous and subject to both analytical and biological limitations. The available studies vary significantly in terms of population, clinical context, biological material (serum, plasma, tissue), assay platform, the selected Elabela isoform, and the reported direction of associations. A structured synthesis is presented in Table 4.

Several patterns emerge. In type 2 diabetes, cross-sectional studies have shown reduced circulating Elabela concentrations compared with controls, with further reductions as diabetic complications progress [18,19,36]. Lower serum Elabela concentrations have been associated with increased albuminuria in diabetic kidney disease [18,19,36], and preliminary data suggest reduced circulating Elabela in proliferative diabetic retinopathy compared with less advanced stages [20]. These studies do not directly address adipocyte lipid metabolism; however, they indicate that Elabela acts as a context-dependent correlate of the burden of diabetic complications.

In obesity itself and classically defined metabolic syndrome, human data remain limited and inconsistent, and the current literature does not allow circulating Elabela to be considered a stable biomarker of these phenotypes. Against this background, the discrepancy between the stronger evidence base for apelin and the weaker evidence for Elabela remains clear [4,6,8,9].

In cardiometabolic populations, lower circulating Elabela concentrations have been reported in patients with hypertension and increased carotid intima-media thickness [17] and in atherosclerotic cohorts, where plasma Elabela correlated negatively with markers of plaque remodelling [21]. Local measurements in epicardial adipose tissue in patients with multivessel coronary artery disease showed reduced tissue Elabela levels associated with higher BMI, total cholesterol and LDL-C [16]. Tissue measurements and those taken in the immediate vicinity of lesions therefore appear to be more informative than isolated circulating values.

A critical methodological caveat applies to this entire body of data. Apart from biological heterogeneity, human Elabela studies are limited at the analytical level: there is no internationally standardised reference method; commercially available ELISAs differ in their capture/detection epitopes and preferred isoforms (ELA-32, ELA-21, ELA-11), with largely uncharacterised analytical specificity and cross-reactivity [14,15,17,18,19,20]. Pre-analytical factors—tube type, anticoagulants, time to processing, freeze–thaw cycles, and matrix type (serum vs. plasma vs. tissue lysate)—may further influence measured concentrations, and matrix effects have not been systematically investigated. Reproducibility between studies and laboratories is therefore limited not only by biological variability but also by analytical incomparability, which in turn limits formal biomarker validation. Several further limitations restrict the translational value of Elabela as a clinical biomarker. There are currently no validated reference ranges for any of the bioactive isoforms (ELA-32, ELA-21, ELA-11) in healthy or metabolically defined populations; inter-laboratory reproducibility has not been formally established; and short peptide biomarkers of this class are intrinsically vulnerable to enzymatic degradation, freeze–thaw instability and matrix-dependent recovery, none of which has been systematically characterised for Elabela. The literature is dominated by single-centre cross-sectional studies, with no published longitudinal cohort tracking circulating concentrations over time, across clinical events or in response to intervention. Plausible confounders—including renal clearance (particularly relevant given the high renal expression of Elabela and its altered concentrations in chronic kidney disease), pharmacological treatment of obesity (including GLP-1 receptor agonists and dual incretin-based therapies), antihypertensive therapy (renin–angiotensin–aldosterone system inhibitors, calcium-channel blockers, β-adrenergic blockade) and lipid-lowering treatment—have not been controlled for or systematically reported. Taken together, these limitations imply that current Elabela measurements should not be regarded as a reliable clinical biomarker, and that any translational use in metabolic medicine is, at present, premature.

An additional interpretative limitation concerns causality. As the available human studies are cross-sectional, they do not allow us to determine whether altered Elabela concentrations are causal factors of the observed phenotype, compensatory responses to metabolic or vascular damage, tissue adaptations, or epiphenomenal signals co-occurring with disease progression without a mechanical contribution. This ambiguity must be accepted as an inherent limitation of the current clinical evidence base [14,15,16,17,18,19,20,21]. Current data support the interpretation of Elabela as a contextual correlate of stress or cardiometabolic adaptation, rather than as a validated standalone biomarker specific to adipose tissue lipid metabolism.

## 10. What Has Been Established for Apelin, and What Cannot Yet Be Assumed for Elabela

Elabela and apelin share APLNR, but differ in receptor engagement, signalling bias, tissue distribution and the weight of experimental evidence [5,22,23,25]. Translational rigour is therefore required whenever data on apelin are cited in the literature on Elabela. Several metabolic effects are well established for apelin, but at present they cannot be assumed for Elabela without additional direct evidence; a comparison is summarised in Table 5.

Regarding lipolysis, apelin-13 modulates lipolytic activity and glycerol release in adipocyte-related models, whilst apelin itself is a classic adipokine up-regulated by insulin and obesity, secreted by mature adipocytes in both humans and mice [9,11,12,13]. Analogous adipocyte data for Elabela are lacking. Regarding glucose homeostasis, apelin improves insulin sensitivity, increases glucose uptake in skeletal muscle and lowers blood glucose levels in numerous rodent models [9,11,12]; the metabolic effects of Elabela in skeletal muscle, liver and adipose tissue remain much less well defined [4,6,8,9]. Regarding hepatic lipid metabolism, apelin has been associated with the modulation of hepatic lipogenesis and steatosis in experimental models [9,11], whilst Elabela-specific data remain limited.

Regarding signalling, even shared APLNR-dependent effects should not be assumed to be equivalent. Elabela and apelin exhibit distinct patterns of receptor binding, internalisation and signalling bias; in particular, ELA-32 exhibits a strong β-arrestin bias in APLNR signalling, whilst apelin peptides differ in their relative engagement of G proteins and β-arrestins [22,23,25]. Under certain experimental conditions, the effects of Elabela do not appear to be fully explained by canonical APLNR engagement [21]. Ligand preferences in specific fat depots or types of immunometabolic cells have not been systematically mapped for Elabela.

Direct extrapolation of metabolic claims derived from apelin to Elabela is therefore not scientifically justified. Any significant claim regarding Elabela in lipid and glucose metabolism should—where possible—be supported by evidence specific to Elabela, and apelin data should serve solely as contextual biological background. This principle is applied throughout the review.

## 11. Elabela at the Crossroads of Cardiometabolic Signalling

The apelinergic system operates at the interface of metabolism, vascular biology and inflammation. Atherosclerotic data provide a strong cardiometabolic dimension: reduced Elabela concentrations correlate with the severity of atherosclerosis, and exogenously administered ELA-21 exhibits anti-inflammatory and anti-atherosclerotic effects under high-fat diet conditions [21]. Tissue observations from epicardial adipose tissue in multivessel coronary artery disease [16] and data on subclinical atherosclerosis in hypertensive cohorts [17] further suggest that local or tissue-specific Elabela patterns may be more informative than circulating values [6]. The overall picture is consistent with a role in the metabolic consequences of impaired lipid metabolism, rather than in the primary, intracellular regulation of triglyceride turnover in the adipocyte.

## 12. Therapeutic Perspective

From a translational perspective, Elabela is biologically interesting but not clinically validated. Its appeal stems from the convergence of several features: anti-inflammatory action, antioxidant and cytoprotective signalling, vascular and endothelial protection, potential modulation of immunometabolic balance, and tissue associations with cardiometabolic burden [8,17,21,24].

The two translational pathways must remain strictly separate. Biomarker utility depends on analytical standardisation—harmonisation of assays with clearly defined epitopes, isoform specification, analytical reproducibility across matrices and laboratories, and long-term clinical validation in well-characterised metabolic populations. Correlational, cross-sectional associations—however promising—do not meet this threshold. Therapeutic utility, however, depends on the confirmation of mechanistic causality (ideally using loss-of-function and gain-of-function strategies), pharmacological accessibility of the receptor (ligand stability, selectivity, acceptable dosage window, safety profile), tissue specificity, and reproducible effects at the intervention level in independent models. Elabela’s biomarker narrative is at an earlier stage of development than the therapeutic narrative, and neither is sufficiently advanced to justify translational-clinical claims.

The development of stabilised peptide analogues with improved bioavailability and receptor-selective agonists is ongoing [8,25,37]. At the present stage, Elabela is best regarded as a candidate indirect modulator of the metabolic stress environment and a candidate biomarker of lipid-related cardiometabolic burden, rather than as a validated master regulator of lipid metabolism. The translational readiness of Elabela should not be overstated. There are currently no validated preclinical metabolic disease models specifically dedicated to Elabela-based therapy, no completed or ongoing clinical intervention trials assessing Elabela analogues in metabolic indications, and no Phase I–II pharmacological data in human metabolic populations. The receptor pharmacology of APLNR engagement by individual Elabela isoforms remains incompletely defined: dose–response relationships, receptor occupancy thresholds, biased-agonism profiles in metabolically relevant cell types, off-target activity, and long-term receptor desensitisation kinetics have not been systematically characterised in the context of metabolic disease. Accordingly, references to therapeutic targeting, biomarker deployment, drug development or clinical translation throughout this review should be understood as describing prospective research directions rather than current translational maturity. Any positioning of Elabela as a clinically actionable target—diagnostic or therapeutic—in lipid-related cardiometabolic disease is, at this stage, premature.

## 13. Limitations of the Current Evidence Base

The literature has several limitations. Direct intra-adipocyte data are scarce (Section 5 and Table 3), and the metabolic evidence base is heavily skewed in favour of apelin rather than Elabela (Section 10 and Table 5). Clinical evidence is mainly cross-sectional and biomarker-based and is further limited analytically by a lack of standardisation of assays, uncharacterised isoform specificity, pre-analytical variability, and limited reproducibility between studies [14,15,16,17,18,19,20,21]. Biological questions also remain unresolved, including the relative roles of individual isoforms, tissue-specific expression patterns, receptor bias, and the extent of Elabela–apelin interactions in APLNR signalling [22,23,25]. This review is narrative rather than systematic in nature (Section 2) and carries the interpretative limitations inherent to this project.

## 14. Directions for Future Research

Several gaps limit the unambiguous assignment of Elabela’s role in metabolic regulation. The central question is whether Elabela acts as a true effector of adipocyte metabolism or primarily reflects tissue stress and broader cardiometabolic adaptation [4,9,13,14].

Future research should move beyond biomarker associations and prioritise direct mechanistic experiments in adipocyte models and adipose tissue—human primary adipocytes, adipocytes derived from the stromal-vascular fraction, and validated mouse models—in combination with loss-of-function and gain-of-function strategies for APLNR. Priority endpoints include lipogenesis, lipolysis, fatty acid oxidation, mitochondrial respiration, glucose uptake, “browning” transcriptional programmes, and interactions with insulin-dependent, AMPK, PI3K/Akt and PPARγ pathways [9,13,35].

The biology of the receptor requires further clarification. Although Elabela is a recognised APLNR ligand, observations from atherosclerosis models—in which the anti-inflammatory effect of ELA-21 was paradoxically enhanced by an APLNR inhibitor—raise the possibility of context-dependent receptor engagement, signalling bias, or non-canonical mechanisms [21,22,23].

Cell type specificity is another priority. Adipocytes, endothelial cells, vascular smooth muscle cells, macrophages and other stromal components may all contribute to the observed phenotype, so co-culture systems, depot-dependent analyses and single-cell transcriptomics approaches will be particularly informative [9,13,21].

A direct comparison of isoforms is also required. The relative metabolic relevance of ELA-32, ELA-21 and ELA-11 remains unresolved, and these isoforms may differ in stability, receptor engagement, signalling bias and tissue distribution [8,22,23,25].

Clinical trials should standardise biological matrices (serum, plasma, tissue), assay platforms and isoform specifications, and move towards long-term studies in well-characterised metabolic populations; such harmonisation is a prerequisite for any meaningful biomarker validation.

Translational work should focus on stabilised analogues, receptor-selective agonists and tissue-targeted delivery strategies—but only after mechanistic uncertainty has been substantially reduced [8,25].

## 15. Conclusions

Elabela is an endogenous ligand of APLNR and an integral component of the apelinergic system. Its roles in embryogenesis, cardiovascular biology, endothelial protection and cytoprotection at the organ level are increasingly well characterised. Its role in metabolic regulation—particularly in adipose tissue and lipid metabolism—remains incompletely defined.

A review of this literature supports a biologically plausible relevance to metabolic homeostasis via APLNR-dependent pathways associated with the control of oxidative stress, modulation of inflammation, cell survival and tissue adaptation [21,34,35]. It also supports a justified role in lipid-related metabolic pathology, particularly in the context of macrophage lipid handling, atherosclerosis and local adipose tissue dysfunction [16,17,21]. Direct evidence that Elabela acts as a primary regulator of adipocyte lipogenesis, lipolysis, fatty acid oxidation or adipocyte differentiation remains insufficient.

Elabela should therefore be regarded primarily as a candidate indirect modulator of metabolic homeostasis and lipid-related dysfunction, and as a candidate biomarker of stress or cardiometabolic adaptation—not as a validated direct regulator of adipocyte lipid metabolism.

Decisive progress will depend on direct studies testing the metabolic effects of Elabela—intra-adipocyte, depot-dependent, isoform-specific, differentiated by cell type and long-term—within its recognised isoforms (ELA-32, ELA-21, ELA-11), anatomically and metabolically distinct adipose tissue depots (subcutaneous, visceral, perivascular, epicardial), and complementary experimental levels (cellular, organoid, in vivo) and—clinically—in prospective long-term cohorts with harmonised analytical methodology.

## Figures and Tables

**Table 1:** Current evidence map regarding the role of Elabela in metabolic regulation.

Area	Current Level of Support	Key Message
Elabela as an APLNR ligand	Strong	Elabela is an endogenous ligand of APLNR; it inhibits cAMP production, activates ERK1/2 and mobilises intracellular Ca^2+^.
Elabela isoforms	Strong	ELA-32, ELA-21 and ELA-11 are recognised isoforms resulting from the processing of the precursor encoded by APELA.
Ligand-specific bias in APLNR signalling	Moderate to strong	Apelinergic ligands activate APLNR via distinct G protein- and β-arrestin-dependent pathways; ELA-32 exhibits a strong β-arrestin bias.
Broad tissue relevance	Strong	Elabela’s biology is well documented in embryonic, cardiovascular, renal, endothelial and tissue-protective contexts.
Direct regulation of adipocyte lipid metabolism	Weak	Current data do not demonstrate robust, reproducible direct control of lipogenesis, lipolysis or fatty acid oxidation by Elabela.
Relevance at the adipose tissue microenvironment level	Moderate	Likely effects may occur via stromal-vascular signalling, at the vessel–adipose tissue and macrophage–adipocyte interfaces, and not solely through lipid turnover in mature adipocytes.
Indirect metabolic relevance	Moderate	Elabela likely modulates oxidative stress, inflammation, mitochondrial function and insulin-like signalling.
Macrophage lipid metabolism	Moderate to strong	ELA-21 inhibits M1 polarisation and oxLDL-induced foam cell formation in atherosclerosis models.
APLNR-independent signalling	Moderate	Some effects may not be entirely APLNR-dependent; alternative receptor mechanisms are possible.
Role as a clinical biomarker	Moderate (analytically limited)	Circulating and tissue concentrations of Elabela may reflect cardiometabolic burden, but variability in assays, isoforms and pre-analytical factors limits comparability.

APELA—the gene encoding Elabela; APLNR—the apelin receptor (also known as APJ); cAMP—cyclic adenosine 3′,5′-monophosphate; ELA—Elabela; ERK1/2—extracellular signal-regulated kinases 1 and 2; M1/M2—pro- and anti-inflammatory macrophage phenotypes; oxLDL—oxidised low-density lipoprotein.

**Table 2:** Priority experimental programme for defining the metabolic role of Elabela.

Research Question	Recommended Model	Main Endpoints	Rationale
Does Elabela directly regulate lipid turnover in adipocytes?	Human primary adipocytes, 3T3-L1, adipocytes from hASC	Lipogenesis, lipolysis, release of FFA and glycerol, phosphorylation of ACC/HSL/ATGL	Central intra-adipocyte question.
Does Elabela modulate the adipose tissue microenvironment?	Ex vivo adipose tissue explants; adipocyte–macrophage and adipocyte–endothelial co-cultures	Cytokines, adipokines, endothelial markers, immune cell polarisation, paracrine signalling	Covers effects in the microenvironment, not just within the adipocyte.
Does Elabela improve mitochondrial health?	Adipocytes with Seahorse measurements	OCR, respiratory reserve, ROS, mitochondrial membrane potential	A mechanistic bridge to the biology of metabolic stress.
Does Elabela influence browning/thermogenesis?	Beige/BAT models, WAT browning pathways	UCP1, PGC-1α, markers of mitochondrial biogenesis	Test of energy dissipation effects.
Is APLNR essential for all effects of Elabela?	APLNR knockout/knockdown and rescue models	cAMP, ERK1/2, Akt, Ca^2+^, β-arrestin recruitment, metabolic endpoints	Distinguishes between canonical and non-canonical signalling.
Are the effects cell- and tissue-specific?	Adipocyte–macrophage–endothelial co-cultures; depot-specific adipose tissues	Cytokines, lipid accumulation, insulin signalling, transcriptomics	Reflects the tissue microenvironment and depot heterogeneity.
Which isoform is metabolically dominant?	Head-to-head ELA-32 vs. ELA-21 vs. ELA-11	Receptor bias, potency, half-life, metabolic effects below	Required for mechanistic and translational clarity.
Can Elabela become a therapeutic platform?	Stabilised analogues, targeted delivery, HFD models	PK/PD, tissue targeting, efficacy, safety	Essential prior to major translational claims.

BAT—brown adipose tissue; FFA—free fatty acid; HFD—high-fat diet; OCR—oxygen consumption rate; PGC-1α—peroxisome proliferator-activated receptor γ co-activator 1α; PK/PD—pharmacokinetics/pharmacodynamics; ROS—reactive oxygen species; UCP1—uncoupling protein 1; WAT—white adipose tissue.

**Table 3:** Direct evidence vs. indirect evidence regarding the metabolic role of Elabela.

Level of Evidence	Directly Demonstrated	Biologically Plausible but Inferred	Hypothetical or Extrapolated
Receptor/ligand-level biology	Elabela binds APLNR; ↓ cAMP, ↑ ERK1/2, ↑ Ca^2+^ mobilisation; ELA-32/21/11 from APELA precursor; ligand-specific bias: G-protein vs. β-arrestin.	Signalling bias likely has functional consequences across cell types.	The metabolic consequences of this bias in adipocytes remain unproven.
Organ-level/tissue-protective effects	Alleviation of oxidative stress, fibrosis, apoptosis and inflammation in cardiac, renal and endothelial models.	Similar effects could occur in adipose tissue under the influence of obesity-related stress.	Direct tissue-protective effects in human adipose tissue have not been demonstrated.
Immunometabolic effects (macrophages)	ELA-21 inhibits M1 polarisation and oxLDL-induced foam cell formation; it restores the M1/M2 balance.	The macrophage effects likely influence lipid-related cardiometabolic damage more broadly.	It has not been established whether immunometabolic effects significantly alter lipid metabolism within the adipocyte.
Effects at the adipose tissue microenvironment level	No direct demonstration in the intact human adipose tissue microenvironment.	Effects on stromal-vascular cells, endothelium and macrophage–adipocyte communication are plausible given the distribution of APLNR.	Whether Elabela significantly remodels the microenvironments specific to fat depots remains unknown.
Direct intra-adipocyte effects	There is no conclusive evidence that Elabela regulates lipogenesis, lipolysis, fatty acid oxidation or differentiation at physiological concentrations.	The expression of APLNR in adipocytes and a shared signalling pathway with apelin make an adipocyte role plausible.	Claims of direct adipocyte control are based on extrapolations from apelin and extra-adipocyte systems.
Biomarker evidence in humans	Reduced circulating and tissue levels of Elabela have been observed in several cardiometabolic populations.	Local or lesion-specific measurements may be more informative than circulating levels.	Cross-sectional associations do not distinguish between causal, compensatory, adaptive and epiphenomenal signals.
Contextual evidence from apelin	Apelin exhibits direct effects on lipolysis, obesity, glucose uptake and liver metabolism.	The shared biology of APLNR suggests possible functional overlap with Elabela.	Extending metabolic conclusions from apelin to Elabela is not justified without direct data.

APELA—the gene encoding Elabela; APLNR—the apelin receptor (APJ); cAMP—cyclic AMP; ELA—Elabela; ERK1/2—extracellular signal-regulated kinases 1 and 2; M1/M2—pro- and anti-inflammatory macrophages; oxLDL—oxidised LDL. Arrows indicate increases (↑) or decreases (↓).

**Table 4:** Summary of human studies on Elabela in obesity, metabolic syndrome, diabetes and lipid-related cardiometabolic disease.

Study/Year [Ref.]	Population	Clinical Context	Material/Measurement	Main Results and Direction	Design and Key Limitations	Main Source of Heterogeneity/Risk of Misinterpretation
Hanssens et al. 2022 [15]	Adults with and without obesity	Obesity	Serum Elabela; ELISA	No consistently robust differentiation between obese and non-obese individuals in standard circulating markers.	Cross-sectional; moderate sample size; single-centre.	Variability in measurements and isoforms; limited phenotypic resolution.
Rachwalik et al. 2025 [16]	Multivessel CAD (*n* = 51) vs. controls (*n* = 34)	Multivessel CAD undergoing revascularisation	Serum + epicardial adipose tissue; ELISA	↓ Elabela in serum and tissue in CAD; lower local Elabela in EAT associated with higher BMI, TC, LDL-C.	Cross-sectional; surgical population; no follow-up.	Mixing of matrices; selection bias in the surgical population.
Hendrianus et al. 2023 [17]	Adults with hypertension (*n* = 104)	Hypertension with subclinical atherosclerosis	Plasma Elabela; ELISA	↓ Circulating Elabela in stage 2 hypertension vs. stage 1 and with ↑ IMT; ↓ Elabela associated with higher CV risk (OR 5.0).	Cross-sectional; single-centre; potential residual bias.	Pre-analytical variability; limited data on BP therapy.
Onalan et al. 2020 [18]	T2DM (*n* = 100) + controls (*n* = 50), ACR stratification	T2DM with diabetic kidney disease	Serum Elabela; ELISA	↓ Elabela in T2DM vs. controls; further ↓ with increasing albuminuria and kidney damage.	Cross-sectional; renal focus; no data on adipose tissue.	The kidney as the predominant ELA-producing organ hinders systemic interpretation.
Zhang et al. 2018 [19]	T2DM stratified by albuminuria	T2DM with albuminuria	Serum Elabela/Toddler; ELISA	Serum Elabela associated with albuminuria; proposed as a predictor in diabetic kidney disease.	Cross-sectional; single population; biomarker-oriented.	Determination of the Toddler isoform not fully characterised analytically.
Seyithanoğlu et al. 2025 [20]	T2DM by DR severity (*n* = 20 each) + controls *n* = 20	T2DM with diabetic retinopathy	Serum Elabela; ELISA	↓ Serum Elabela with increasing severity of retinopathy; preliminary biomarker signal.	Pilot cross-sectional study; small subgroups; requires replication.	Effects of small sample size; validation of a single ELISA kit not independently confirmed.
Tang et al. 2025 (human cohort) [21]	Atherosclerosis vs. controls	Atherosclerosis	Elabela in plasma; ELISA	↓ Elabela in plasma in patients with atherosclerosis; negative correlation with MMP2 and MMP9.	Cross-sectional clinical component of a mixed study.	Mixed-methods study; the clinical arm is limited in terms of sample size and phenotypic depth.

Interpretative note: The summarised studies encompass clinically heterogeneous populations, different biological matrices (serum, plasma, tissue), different ELISA platforms with uncharacterised isoform specificity, and variable pre-analytical procedures. Cross-sectional associations do not allow us to determine whether Elabela changes reflect causal, compensatory, adaptive or epiphenomenal processes. The table should be read as a map of heterogeneity rather than as a homogeneous body of evidence supporting a single unified biological conclusion. ACR—urine albumin-to-creatinine ratio; BMI—body mass index; BP—blood pressure; CAD—coronary artery disease; CV—cardiovascular; DR—diabetic retinopathy; EAT—epicardial adipose tissue; ELISA—enzyme-linked immunosorbent assay; IMT—intima-media thickness; LDL-C—low-density lipoprotein cholesterol; MMP—matrix metalloproteinase; OR—odds ratio; TC—total cholesterol; T2DM—type 2 diabetes. Upward and downward arrows indicate increases and decreases, respectively.

**Table 5:** What has been established for apelin, and what cannot yet be assumed for Elabela.

Area	Established for Apelin (Selected)	Currently Not Validated/Insufficient for Elabela
Adipocyte lipolysis and adipokine status	Apelin is a classic adipokine secreted by mature human and mouse adipocytes, up-regulated by insulin and obesity [11,12,13]; apelin-13 modulates lipolytic activity and glycerol release.	Elabela is not classified as a classic adipokine; lack of robust adipocyte-derived lipolytic/antilipolytic data; secretion and regulation in adipose tissue insufficiently characterised.
Hepatic lipid metabolism	Apelin has been experimentally linked to the modulation of hepatic lipogenesis and steatosis [9,11].	Direct data on hepatic lipid metabolism specific to Elabela are limited.
Glucose homeostasis and insulin sensitivity	Apelin increases glucose uptake in skeletal muscle, improves insulin sensitivity and lowers blood glucose levels in rodent models [9,11,12].	Systematic data specific to Elabela regarding glucose homeostasis in skeletal muscle, liver and adipose tissue are scarce [4,6,8,9].
Effects on obesity and body weight	Apelin influences obesity-related endpoints in rodent studies [11,12].	No analogous fat-specific effects on body weight or fat deposits for Elabela have been established in mechanistic models.
APLNR engagement and signalling bias	Apelin-driven APLNR signalling is extensively characterised, including Gi and β-arrestin pathways [5,22].	Elabela exhibits distinct receptor engagement and a strong β-arrestin bias (ELA-32) compared to apelins [22,23]; metabolic consequences remain unclear.
Circulating biomarker profile	Apelin concentrations vary with obesity, diabetes, weight loss and glycaemic control [11,12,14].	Biomarker data for Elabela are less abundant, more heterogeneous in terms of assays, and limited in long-term studies [14,15,17,18,19,20].
Translational/therapeutic maturity	Stabilised apelin analogues and small-molecule APLNR agonists are more advanced in the translational pipeline [11,12,24].	Elabela analogues are under development [8,25], but remain at earlier stages; metabolic indications have not yet been validated.

The biology of apelin should be treated as contextual background for Elabela, rather than as direct evidence of its action. Significant metabolic claims regarding Elabela should be supported, where possible, by experimental data specific to Elabela.

## Data Availability

No new data were generated or analysed as part of this study. All cited sources are publicly available in the original journals listed in the references.
